# Supplementary material for: An approach to increase the power output of Karnafuli Hydroelectric Power Station: A step to sustainable development in Bangladesh’s energy sector
Source: PLoS One. 2021 Oct 11;16(10):e0257645. doi: 10.1371/journal.pone.0257645 (PMC8504746; doi:10.1371/journal.pone.0257645)
Supplement: Attachment — Submitted filename: Response_to_Reviewers.pdf [file pone.0257645.s001.pdf]

A rebuttal letter

Response to Reviewers

Editor Comments:

1. Please ensure that your manuscript meets PLOS ONE's style requirements, including those for file naming. The PLOS ONE style templates can be found at

https://journals.plos.org/plosone/s/file?id=wjVg/PLOSOne_formatting_sample_main_body.pdf and

https://journals.plos.org/plosone/s/file?id=ba62/PLOSOne_formatting_sample_title_authors_affiliations.pdf

Addressed:

Thank you. We have followed the style according to the style format of PLOS ONE. We used the Template for PLoS-Version 3.5 March 2018 Latex version.

2. PLOS requires an ORCID iD for the corresponding author in Editorial Manager on papers submitted after December 6th, 2016. Please ensure that you have an ORCID iD and that it is validated in Editorial Manager. To do this, go to 'Update my Information' (in the upper left-hand corner of the main menu), and click on the Fetch/Validate link next to the ORCID field. This will take you to the ORCID site and allow you to create a new iD or authenticate a pre-existing iD in Editorial Manager. Please see the following video for instructions on linking an ORCID iD to your Editorial Manager account: https://www.youtube.com/watch?v=_xcclfuvtxQ

I already have an ORCID ID.

3. Please amend the manuscript submission data (via Edit Submission) to include authors Tahmidur Rahman, Abdullah Al Mamun, Omar Bin Mannan.

Addressed: We have included the co-authors.

4. We note that Figure1 in your submission contain map/satellite images which may be copyrighted. All PLOS content is published under the Creative Commons Attribution License (CC BY 4.0), which means that the manuscript, images, and Supporting Information files will be freely available online, and any third party is permitted to access, download, copy, distribute, and use these materials in any way, even commercially, with proper attribution. For these reasons, we cannot publish previously copyrighted maps or satellite images created using proprietary data, such as Google software (Google Maps, Street View, and Earth). For more

information, see our copyright guidelines: http://journals.plos.org/plosone/s/licenses-and-copyright.

We require you to either (1) present written permission from the copyright holder to publish these figures specifically under the CC BY 4.0 license, or (2) remove the figures from your submission:

4.1. You may seek permission from the original copyright holder of Figure 1 to publish the content specifically under the CC BY 4.0 license.

We recommend that you contact the original copyright holder with the Content Permission Form (http://journals.plos.org/plosone/s/file?id=7c09/content-permission-form.pdf) and the following text:

"I request permission for the open-access journal PLOS ONE to publish XXX under the Creative Commons Attribution License (CCAL) CC BY 4.0 (http://creativecommons.org/licenses/by/4.0/). Please be aware that this license allows unrestricted use and distribution, even commercially, by third parties. Please reply and provide explicit written permission to publish XXX under a CC BY license and complete the attached form."

Please upload the completed Content Permission Form or other proof of granted permissions as an "Other" file with your submission.

In the figure caption of the copyrighted figure, please include the following text: "Reprinted from [ref] under a CC BY license, with permission from [name of publisher], original copyright [original copyright year]."

4.2. If you are unable to obtain permission from the original copyright holder to publish these figures under the CC BY 4.0 license or if the copyright holder's requirements are incompatible with the CC BY 4.0 license, please either i) remove the figure or ii) supply a replacement figure that complies with the CC BY 4.0 license. Please check copyright information on all replacement figures and update the figure caption with source information. If applicable, please specify in the figure

caption text when a figure is similar but not identical to the original image and is therefore for illustrative purposes only.

The following resources for replacing copyrighted map figures may be helpful:

USGS National Map Viewer (public domain): http://viewer.nationalmap.gov/viewer/

The Gateway to Astronaut Photography of Earth (public domain): http://eol.jsc.nasa.gov/sseop/clickmap/

Maps at the CIA (public domain): https://www.cia.gov/library/publications/the-world-factbook/index.html and https://www.cia.gov/library/publications/cia-maps-publications/index.html

NASA Earth Observatory (public domain): http://earthobservatory.nasa.gov/

Landsat: http://landsat.visibleearth.nasa.gov/

USGS EROS (Earth Resources Observatory and Science (EROS) Center) (public domain): http://eros.usgs.gov/#

Natural Earth (public domain): http://www.naturalearthdata.com/

Addressed:

We are extremely sorry for the copy right issue. We have tried to take the Google permission but it seemed to taking time. We redraw the Figure-1 in Illustrator to display the site of the KH plant.

5. Please include a copy of Table 6 which you refer to in your text on page 10.

Addressed:

Thank you for the comment. It was a typo, sorry. It will be Table-3. We have corrected it.

6. Please upload a copy of Supporting Information Figure/Table/etc. xxxx which you refer to in your text on page xx.

Addressed:

The supporting information that presented in the manuscript were the same Figures and Tables in the manuscript. Therefore, no additional data or information remains. We have omitted this part from the manuscript.

Reviewers' comments:

Reviewer's Responses to Questions

Comments to the Author

1. Is the manuscript technically sound, and do the data support the conclusions?

The manuscript must describe a technically sound piece of scientific research with data that supports the conclusions. Experiments must have been conducted rigorously, with appropriate controls, replication, and sample sizes. The conclusions must be drawn appropriately based on the data presented.

Reviewer #1: Partly

Reviewer #2: Partly

Ans: Thank you for the comments.

---

2. Has the statistical analysis been performed appropriately and rigorously?

Reviewer #1: N/A

Reviewer #2: No

Ans: Thank you for comments. We have data collection phase and simulation results to show the consistency of the existing KH plant output compared to our estimation theoretically.

---

3. Have the authors made all data underlying the findings in their manuscript fully available?

The PLOS Data policy requires authors to make all data underlying the findings described in their manuscript fully available without restriction, with rare exception (please refer to the Data Availability Statement in the manuscript PDF file). The data should be provided as part of the manuscript or its supporting information, or deposited to a public repository. For example, in addition to summary statistics, the data points behind means, medians and variance measures should be available. If there are restrictions on publicly sharing data—e.g. participant privacy or use of data from a third party—those must be specified.

Reviewer #1: No

Reviewer #2: No

Ans: Thank you for comments. Actually we have provided all data and information in the corresponding Tables and Figures. We mistakenly added some supporting information data, but those are same figures and Tables in the manuscript. Therefore, all data are within the manuscript. Sorry for the inconvenience.

---

4. Is the manuscript presented in an intelligible fashion and written in standard English?

PLOS ONE does not copyedit accepted manuscripts, so the language in submitted articles must be clear, correct, and unambiguous. Any typographical or grammatical errors should be corrected at revision, so please note any specific errors here.

Reviewer #1: No

Reviewer #2: Yes

Ans: Thank you for comments. We have rewritten the manuscript thoroughly and added more sections to present in more readable form. We added subsection 1.1 in page-2 under the title of related works and Motivation.

---

5. Review Comments to the Author

Please use the space provided to explain your answers to the questions above. You may also include additional comments for the author, including concerns about dual publication, research ethics, or publication ethics. (Please upload your review as an attachment if it exceeds 20,000 characters)

Reviewer #1:

The first and general comment relates to the written form, as the paper requires a serious improvement in English grammar and spelling. The English should be

improved. A deep proofreading is needed, many errors can be seen through the document.

Addressed: Thank you for your comment. We have checked the manuscript thoroughly and try to improve the writing. The proofread has performed in peer.

ii)The introduction and the related review of the literature is poor provided. And the structure must be improved for a better understanding of the current state of the art. Also, the drawbacks of the existing methods must be highlighted clearly for justifying the upgrade proposed by the current work. The way the latter work is improving the state of the art must be clarified.

Address: We have rewritten the Introduction section. We added an extra subsection named "1.1 Related works and Motivation" under the Introduction Section on page-2, in line-40. We have rearranged the structure of the manuscript so that it may understandable more.

iii) Novelty of the paper is not mentioned obviously. There're a grammatical and syntax errors.

Address: Thank you for the comments. We have rewritten the Introduction section so that the novelty of this work is focused. All grammatical and syntax errors are revised in a peer review process. The motivation subsection in line-40 may display the novelty of our proposed method. We have added 12 more recent papers and sited them to show the novelty of our work.

iv) Authors are encouraged to introduce a nomenclature section at the beginning of the manuscript, including all variables, acronyms, indexes and constants defined in the manuscript, in order to make the text more clear and readable.

Address: Thank you so much for the comments. A nomenclature in item formatted has been introduced in Section-3 from lines 186-203.

v) In the introduction section, the authors need more to introduce the previous literature. A comprehensive paper needs more than 40 references at least.

Address: We have rewritten the Introduction section and accommodated 12 more papers from previous literatures. We added an extra subsection named "Related works and Motivation" under the Introduction Section (line 39 to 106). We have cited more relevant papers from the recent publications. New references are [24-25], [30-35] and [38-41].

vi) Figures should be given with better accuracy and described in the paper. Figures must be replaced with high resolution ones.

Address: We have simulated all results with better accuracy. Some of them are redrawn with illustrator for better dpi. Figures are replaced with higher resolutions. The redrawn figures are Fig-1, Fig-5, Fig-6, Fig-8, Fig-9, and Fig-10.

vii) Authors must be talk about the future work and potential limitations briefly in the Conclusions and Recommendations section.

Address: Thank you for the comments. We have changed the section name from "Conclusions" to "Conclusion and Recommendations". We added future scope from lines 463-472.

viii) Variables in the text must be italic.

Address: All variables in the manuscript are changed to italic form.

iX) All those comments are unfortunate when we see the quality of the numerical results. Via those results, the proposed method demonstrates its effectiveness without any doubt. However, the materials for introducing the state of the art and the methodology is deficient and poor.

Address: Thank you for the comment. We have tried our best to reflect the state of the art by rewriting the introduction section, adding the nomenclatures of different variables, redrawing some figures and citing 12 more papers. Fig-1, Fig-8, Fig-10 and Table-3 are updated. We have added Table-4 for a comparison to practical, simulated and existing results.

<u>Reviewer #2:</u> An Approach to Increase the Power Output of Karnafuli Hydroelectric Power Station: A Step to Sustainable Development in Bangladesh's Energy Sector Although it was felt that your manuscript was quite interesting. The main problems with the paper are as follow:

1-First paragraph of the introduction needs to be rewritten. (You can omit this paragraph)

Address: Thank you so much for the comment. We have omitted this paragraph from the Introduction Section.

2-"In this work, a method is proposed to obtain additional power from the existing plant utilizing the Combined-Cycle Hydropower System (CCHS) technology to increase overall plant capacity [12]." is it done in reference [12]?!

Address: Thank you for the comment. Mistakenly, we have cited a review paper [12] in the Introduction section in lines 40-43. Sorry for the mistake. We have omitted this reference from this section and can be seen from lines 106-107.

3-Introduction needs to be rewritten. Please review more papers and mention their method weaknesses.

Address: We have rewritten the Introduction section and cited 12 more papers from previous literatures. We added an extra subsection named "Related works and Motivation" under the Introduction Section (line 39 to 106). We have cited more relevant papers from the recent publications and displayed their limitation and scope of improvements. New references are [24-25], [30-35] and [38-41].

4-"An Approach to Increase the Power Output of Karnafuli Hydroelectric Power Station" How much did it increase?

Address: Thank you for the comments. It was mentioned (12.2 MW) in the "Simulation Result" Section 6.2 at line 389, unfortunately, we had a wrong referencing Table-6 for comparison. There was no Table-6 in the manuscript, however it should be Table-3. Sorry for the mistake. Now we add this value in Table-3 and compared the value with the estimated (13.32 MW) from surveyed data from the plant in line 390.

5-The main concern with this paper is the contribution of the paper. The mentioned contributions cannot be new idea.

Address: Thank you for the comment. We agreed with you. However, the rewritten Introduction section may reflect our contribution now. The previous works are based on river natural water current. Our contributions are:

- **Real data from the plant: Survey in Kaptai hydropower plant to collect data. We collect the real data of the KH plant.**

- **Analysis to data: Primary analysis and power estimation are performed based on surveyed data. A simulation model is established to analyze the existing KH plant.**
- **Site Selection: There were 4 regions and we have selected the optimum one with proper reasoning.**
- **Combined Technology: CCHS technology is old idea, but we apply it in the plant with analysis and site selection process. Based on the analysis, we proposed the suitable turbine to generate 27% more electricity from the plant.**
- **A prototype model: To verify the proposed model we built a pilot project in a small scale and compared with the simulated results.**

6-Studies have been performed on the Karnafuli Hydroelectric Power Station. Please compare simulation results with practical results (if possible).

Address: Thank you for the comment. We have added Table-4 in section 6.4 "Discussion and Comparison" showing a comparison among the estimated, simulated and experimental results. Lines 434-437 are added to the section to show the comparison.

Table 4. A comparison among estimated, simulated and experimental results.

Parameters	Simulated<br><br>(Unit-4 of KH)	Estimated<br><br>(from Data)	Simulated (proposed)	Prototype<br><br>Model
Voltage	100kV	98 kV	7 kV	2.9 V
Current	190A	151 A	1500A	324 mA
Frequency	50 Hz	50 Hz	50 Hz	50 Hz
Average, P	17.1 MW	13.32 MW	12.20 MW	0.78 Watt

-References should be written as standard

Address: All references are rechecked and maintained with journal standard and Plos One standard.

-In addition, proofread the text to avoid fragments, compliance with English grammar and punctuations rules and subject and verb agreement.

Address: Thank you for the comment. We have double checked the manuscript thoroughly.
